# eNOS genotype modifies the effect of leisure-time physical activity on serum triglyceride levels in a Japanese population

**DOI:** 10.1186/1476-511X-11-150

**Published:** 2012-11-05

**Authors:** Takahiro Higashibata, Nobuyuki Hamajima, Mariko Naito, Sayo Kawai, Guang Yin, Sadao Suzuki, Yoshikuni Kita, Hideshi Niimura, Takeshi Imaizumi, Keizo Ohnaka, Kokichi Arisawa, Masako Shigeta, Hidemi Ito, Haruo Mikami, Michiaki Kubo, Hideo Tanaka, Kenji Wakai

**Affiliations:** 1 Department of Preventive Medicine, Nagoya University Graduate School of Medicine, 65 Tsurumai-cho, Showa-ku, Nagoya, 466-8550, Japan; 2 Department of Public Health, Nagoya City University Graduate School of Medical Sciences, Nagoya, 467-8601, Japan; 3 Department of Health Science, Shiga University of Medical Science, Otsu, 520-2192, Japan; 4 Department of International Island and Community Medicine, Kagoshima University Graduate School of Medical and Dental Science, Kagoshima, 890-8544, Japan; 5 Department of Preventive Medicine, Faculty of Medicine, Saga University, Saga, 849-8501, Japan; 6 Department of Geriatric Medicine, Graduate School of Medical Sciences, Kyushu University, Fukuoka, 812-8582, Japan; 7 Department of Preventive Medicine, Institute of Health Biosciences, the University of Tokushima Graduate School, Tokushima, 770-8503, Japan; 8 Department of Epidemiology for Community Health and Medicine, Kyoto Prefectural University of Medicine, Kyoto, 602-8566, Japan; 9 Division of Epidemiology and Prevention, Aichi Cancer Center Research Institute, Nagoya, 464–8681, Japan; 10 Division of Cancer Registry, Prevention and Epidemiology, Chiba Cancer Center, Chiba, 260-8717, Japan; 11 Laboratory for Genotyping Development, Center for Genomic Medicine, RIKEN, Yokohama, 230-0045, Japan

**Keywords:** Cross-sectional study, Gene-environment interaction, Hypertriglyceridemia, Lifestyle-related disease, *NOS3*, Tailoring prevention

## Abstract

**Background:**

Nitric oxide is a key molecule not only in the cardiovascular system, but also in the metabolic-endocrine system. The purpose of this study was to examine possible associations of the *NOS3* T-786C polymorphism (rs2070744) with serum lipid levels on the basis of lifestyle factors for tailoring prevention of dyslipidemia.

**Methods:**

For this cross-sectional study, a total of 2226 subjects aged 35 to 69 years (1084 men and 1142 women) were selected from Japanese participants in the Japan Multi-Institutional Collaborative Cohort (J-MICC) Study. They were recruited in eight areas throughout Japan between February 2004 and November 2008.

**Results:**

In a stratified analysis by leisure-time physical activity, the likelihood of hypertriglyceridemia (serum triglyceride levels ≥ 150 mg/dL) among subjects with the C allele was significantly lower than those without it in the active group (OR = 0.43, 95% CI = 0.22-0.84 in the fasting group), but not in the sedentary group. A gene-environment interaction between the T-786C polymorphism and leisure-time physical activity for hypertriglyceridemia was significant (*P* = 0.007 in the fasting group). Additionally, serum triglyceride levels (mean ± SD) across leisure-time physical activity classes decreased significantly only in the TC + CC genotype group (111 ± 60 mg/dL for sedentary, 95 ± 48 mg/dL for moderately active, 88 ± 44 mg/dL for very active, *P* for trend = 0.008 in the fasting group), but not in the TT genotype group. Total cholesterol, high-density lipoprotein (HDL) cholesterol, and non-HDL cholesterol levels had no significant association with the polymorphism.

**Conclusions:**

This study suggests that the *NOS3* T-786C polymorphism modifies the effect of leisure-time physical activity on serum triglyceride levels.

## Introduction

Nitric oxide (NO) is an endothelium-derived relaxing factor, and plays a crucial role in the cardiovascular system [1]. NO is synthesized from L-arginine by the nitric oxide synthase (NOS) [2]. NOS consists of three major isoforms: neuronal NOS, inducible NOS, and endothelial NOS (eNOS) [2]. eNOS is encoded by *NOS3*, a gene localized on chromosome 7q35-36 [3], and is constitutively expressed in vascular endothelium, blood platelets, and cardiomyocytes [4]. eNOS-derived NO is involved in regulation of vascular tone and regional blood flow, suppression of vascular smooth muscle proliferation, and inhibition of monocyte adhesion and platelet aggregation [5].

Recently, attention has been focused on eNOS-derived NO as a key molecule, not only in the cardiovascular system, but also in the metabolic-endocrine system [5]. Homozygote eNOS-deficient mice have been shown to be hypertensive and have fasting hyperinsulinemia, hyperlipidemia, and a 40% lower insulin-stimulated glucose uptake than control mice [6]. Similarly, a number of epidemiological studies have shown associations between *NOS3* polymorphisms and endocrine-metabolic disorders. Of these polymorphisms, the *NOS3* T-786C polymorphism, a thymidine to cytosine transition mutation, substantially reduces the promoter activity of *NOS3 in vitro*, resulting in a decrease of endothelial NO production [7]. A case–control study reported that significantly more hypertensive patients with metabolic syndrome were homozygous for the C allele than those patients without it [8].

To date, several previous studies have recruited participants from the healthy population (n = 310, 233, 142, 118, and 101, respectively) to investigate the relationship between the T-786C polymorphism and serum lipid levels [9-13]. However, there has been no large-scale population-based study that has analyzed the association. Provided that gene-environment interactions between the T-786C polymorphism and lifestyle factors on serum lipid levels are well established, the polymorphism may be useful for tailoring prevention of dyslipidemia by lifestyle modification.

Therefore, the purpose of the present study was to examine possible associations of the T-786C polymorphism with serum lipid levels on the basis of lifestyle factors such as obesity, smoking, and leisure-time physical activity, using cross-sectional data from the Japan Multi-Institutional Collaborative Cohort (J-MICC) Study. The study was launched in 2005 to examine gene-environment interactions in lifestyle-related diseases, especially cancers, by 10 research teams throughout Japan [14].

## Materials and methods

### Study subjects

From 4519 subjects recruited between February 2004 and November 2008 in this cross-sectional study [15], subjects who met any of the following criteria were excluded: (i) absence of data about serum lipid levels, physical examination, lifestyle, DNA genotyping, and blood sampling time from their last meal (n = 1416); (ii) medical history of diabetes mellitus, liver cirrhosis, thyroid disorder, nephrotic syndrome, and Cushing syndrome (n = 459); (iii) medication that affects lipid levels (statins, fibrates, β-blockers, steroids, estrogen preparations, and antiestrogens) (n = 227); (iv) blood samples taken within two hours from their last meal (n = 184); (v) living in enrollment areas where there were no subjects with hypertriglyceridemia in stratified analysis, making adjustment for enrollment area impossible (n = 7). The final study group was a total of 2226 subjects (1084 men and 1142 women) from eight areas (Shizuoka, Aichi, Shiga, Kyoto, Tokushima, Fukuoka, Saga, and Kagoshima) throughout Japan. Subjects who fasted for at least 12 hours after their last meal were defined as fasting. All subjects included in this study provided written informed consent, and the J-MICC study including the current study was approved by the ethics committee of Nagoya University School of Medicine (approval No. 253 and 939).

### Clinical and laboratory data

At the time of enrollment, anthropometric and blood pressure measurements, blood samples, and questionnaires were collected from each subject. BMI was calculated on the basis of measured height and body weight at each enrollment area. Blood samples were obtained for analysis of total cholesterol, high-density lipoprotein (HDL) cholesterol and triglycerides by a standard laboratory protocol used in each research team. Non-HDL cholesterol levels were calculated as total cholesterol minus HDL cholesterol levels.

### Lifestyle factors

The questionnaires investigated lifestyle factors such as eating habits, smoking status, and leisure-time physical activity. Eating habits were assessed using a validated food frequency questionnaire [16]. Fat energy ratio was calculated by using the following formula: 9 × fat intake (grams/day)/caloric intake (kcal/day) × 100.

Smoking habits were assessed by smoking status and cumulative exposure. Smoking status included; never, former, and current smoking. In former and current smokers, cumulative smoking exposure was quantified in terms of pack-years by multiplying the number of years smoked by the average number of packs per day. According to pack years, smokers were classified into three levels: light (< 20 pack-years), moderate (< 40 but ≥ 20 pack-years), and heavy (≥ 40 pack-years).

Leisure-time physical activity was evaluated using the International Physical Activity Questionnaire (IPAQ) [17]. IPAQ was developed and validated as an instrument for cross-national monitoring of physical activity and inactivity [17]. From the type, frequency of, and time spent on physical activity during leisure time, the degree of leisure-time physical activity was calculated as metabolic equivalent (MET)-minutes/week (MET levels × minutes of activity/day × days/week) according to IPAQ. The degree of leisure-time physical activity in subjects was classified into three groups: sedentary (< 600 MET-minutes/week), moderately active (< 1500 but ≥ 600 MET-minutes/week), and very active (≥ 1500 MET-minutes/week). In addition, subjects whose leisure-time physical activity was 600 MET-minutes/week or more were defined as active.

### *NOS3* genotyping

Each genomic DNA sample was prepared from peripheral blood by a BioRobot M48 Workstation (Qiagen Group, Tokyo, Japan) or an automatic nucleic acid isolation system (NA-3000, Kurabo, Co., Ltd, Osaka, Japan). The T-786C polymorphism was genotyped by a multiplex polymerase chain reaction (PCR)-based Invader assay (Third Wave Technologies, Madison, WI, USA) at the Laboratory for Genotyping Development, Center for Genomic Medicine, RIKEN, as described previously [18].

For multiplex PCR, a 180-bp fragment encompassing the T-786C polymorphism was amplified with the following primers: 5^′^-TGAAGTGCCTGGAGAGTGC-3^′^ and 5^′^-CCCACCCTGTCATTCAGTG-3^′^. The amplified fragment was diluted and used for the Invader assay. Signal intensities that discriminated genotypes were recorded after the reaction using the ABI7900HT sequence detection system (Applied Biosystems, Foster City, CA, USA).

### Statistical analysis

Chi-squared tests were performed to analyze deviation of observed genotype frequencies from the Hardy-Weinberg equilibrium. Differences in the quantitative data among the genotypes were examined by one-way ANOVA. Normality was tested by the skewness and kurtosis test, and parameters without normal distribution were analyzed after log-transformation.

Odds ratios (ORs) and 95% confidence intervals (CIs) for the presence of the C allele for hypertriglyceridemia (serum triglyceride levels ≥ 150 mg/dL) and *P* values for the interaction between the polymorphism and lifestyle factors for hypertriglyceridemia were calculated by multiple logistic regression analysis, and adjusted in the following two ways: (model 1) for age (continuous), sex (male and female), and enrollment area (Shizuoka, Aichi, Shiga, Kyoto, Tokushima, Fukuoka, Saga, and Kagoshima); (model 2) for age, sex, enrollment area, BMI (continuous), fat energy ratio (continuous), smoking habits (never, light, moderate, and heavy), leisure-time physical activity (sedentary, moderately active, and very active), and blood sampling time from last meal (≥ 12 h, 8 h to < 12 h, 4 h to < 8 h, and 2 h to < 4 h). A multiplicative interaction term was included in the logistic model to test the interactions. The statistical power among total subjects to detect an OR of 0.50, 0.60, or 0.70 for carriers of the C allele was estimated to be 99%, 96%, or 73%, respectively (estimated minor allele carriers frequency in a Japanese population without hypertriglyceridemia = 0.20). Similarly, the powers among the subgroups examined were 98%, 83%, or 53% for BMI < 25, 74%, 50%, or 27% for BMI ≥ 25, 97%, 83%, or 53% for pack-years < 20, 73%, 49%, or 27% for pack-years ≥ 20, 96%, 80%, or 50% for sedentary, and 82%, 58%, or 32% for active, respectively. In the subgroups stratified by leisure-time physical activity, our sample size could detect an OR of 0.50 with more than 80% power.

In addition, serum triglyceride levels were compared between leisure-time physical activity classes, namely, sedentary, moderately active, and very active. Trends of serum triglyceride levels across the classes and differences in serum triglyceride levels between the classes were tested using multiple linear regression models by coding 1, 2, or 3 for sedentary, moderately active, and very active, respectively, as an independent variable with the same covariates as used in model 2 above. The dependent variable was the log-transformed value of serum triglyceride levels. A two-tailed *P* value of < 0.05 was considered to be statistically significant. All analyses were conducted using Stata version 11.0 (Stata Corp., College Station, TX, USA).

## Results

### Frequencies of the *NOS3* T-786C polymorphism

A total of 19.2% of subjects were carriers of the C allele (TC + CC), with an allelic frequency of 10.2%. The T-786C genotype distribution of all subjects followed the Hardy-Weinberg equilibrium (χ^2^ = 0.47, *P* = 0.49, Table 1).

**Table 1:** Characteristics of study subjects by sex

	**Male**	**Female**	**Total (n=2226)**
	**(n=1084)**	**(n=1142)**	
*NOS3* T-786C genotype			
TT	869 (80.2)	930 (81.4)	1799 (80.8)
TC	202 (18.6)	199 (17.4)	401 (18.0)
CC	13(1.2)	13(11)	26(1.2)
Age (y)			
35 to < 40	47(4.3)	54(4.7)	101 (4.5)
40 to < 50	222 (20.5)	236 (20.7)	458 (20.6)
50 to < 60	354 (32.7)	438 (38.4)	792 (35.6)
≥ 60	461 (42.5)	414 (36.3)	875 (39.3)
BMI (kg/m^2^)			
< 25	749 (69.1)	896 (78.5)	1645 (73.9)
25 to < 30	303 (28.0)	212 (18.6)	515 (23.1)
≥ 30	32(3.0)	34 (3.0)	66(3.0)
Fat energy ratio (%)			
< 20	584 (53.9)	151 (13.2)	735 (33.0)
20 to < 25	330 (30.4)	364 (31.9)	694 (31.2)
25 to < 30	123 (11.4)	369 (32.3)	492 (22.1)
≥ 30	47 (43)	258 (22.6)	305 (13.7)
Smoking habits (pack-years)			
Never	333 (30.7)	1046 (91.6)	1 379 (62.0)
Light (< 20)	245 (22.6)	55 (4.8)	300 (13.5)
Moderate (20 to < 40)	283 (26.1)	35 (3.1)	318 (14.3)
Heavy (≥ 40)	223 (20.6)	6 (0.5)	229 (10.3)
Leisure-time physical activity			
(MET-minutes/week)			
Sedentary (< 600)	644 (59.4)	705 (61.7)	1 349 (60.6)
Moderately active (600 to < 1500)	295 (27.2)	305 (26.7)	600 (27.0)
Very active (≥ 1500)	145 (1 3.4)	132 (11.6)	277 (12.4)

Values are presented as n (%).

MET, metabolic equivalent.

### Clinical characteristics of subjects

The characteristics of study subjects by sex are shown in Table 1. Regarding leisure-time physical activity, males and females showed a similar distribution. Table 2 summarizes the BMI, blood pressure, and laboratory parameters of all subjects by the T-786C genotype. Serum triglyceride levels were significantly different among the three genotypes. Total cholesterol, HDL cholesterol, and non-HDL cholesterol had no significant association with the polymorphism.

**Table 2:** **BMI, BP, and laboratory parameters of study subjects by the *NOS3 *T-786C genotype**

**Parameters**	**TT (n= 1799)**	**TC (n= 401)**	**CC (n= 26)**	***P *value**^**a**^
BMI (kg/m^2^)	23 (21–25)	23 (21–25)	24 (20–26)	0.70
Systolic BP (mm Hg)	126 (113–138)	126 (112–138)	126 (116–138)	0.99
Diastolic BP (mm Hg)	78 (70–85)	78 (70–85)	79 (70–84)	0.99
Total cholesterol (mg/dL)	212 (188–234)	207 (187–235)	207 (182–238)	0.63
HDL cholesterol (mg/dL)	62 (52–74)	62 (52–75)	61 (53–73)	0.57
Non-HDL cholesterol (mg/dL)	147 (125–171)	144 (125–169)	147 (114–175)	0.50
Triglycerides (mg/dL)	99 (71–146)	93 (69–135)	92 (65–193)	0.04
Glucose (mg/dL)^b^	94 (89–102)	94 (90–101)	93 (87–100)	0.80
HbA1c (%)^b^	5.1 (4.9-5.3)	5.1 (4.9-5.3)	5.0 (4.8-5.2)	0.75
AST (U/L)	21 (18–25)	21 (17–25)	20 (17–24)	0.17
ALT (U/L)	19 (14–25)	18 (14–25)	1 7 (14–25)	0.56
GGT (U/L)	23 (15–41)	25 (17–39)	24 (16–37)	0.72
Creatinine (mg/dL)	0.76 (0.63-0.90)	0.77 (0.61-0.90)	0.75 (0.67-0.82)	0.90
Uric acid (mg/dL)^b^	5.1 (4.2-6.0)	5.2 (4.2-6.1)	5.0 (3.9-6.3)	0.97

Values are presented as median (interquartile range).

ALT, alanine aminotransferase; AST, aspartate aminotransferase; BP, blood pressure; GGT , gamma-glutamyl transpeptidase; HbA1c, glycosylated hemoglobin; HDL, high-density lipoprotein.

^a^ Analyses were done after log-transformation.

^b^ Data were not available; 391 with TT, 73 with TC, and 6 with CC for glucose, 333, 50, and 5 for HbA1c, respectively, and 1 with TT for uric acid.

### Stratified analysis by lifestyle factors for hypertriglyceridemia

Multiple logistic regression analysis of an association between the T-786C polymorphism and hypertriglyceridemia (serum triglyceride levels ≥ 150 mg/dL) demonstrated that the ORs for the presence of the C allele for hypertriglyceridemia in both the total and fasting group were not significant (Table 3).

**Table 3:** **The *NOS*3 T-786C polymorphism and hypertriglyceridemia (serum triglyceride levels ≥ 150)**

	**TC+CC)vs. TT, model**^**a**^**1**			**(TC+CC)vs. TT, model**^**b**^**2**		
	**OR**	**95%CI**	**Interaction**	**OR**	**95%**	**Interaction**
			***P *value**			***P *value**
Total (n = 2226)	0.80	0.61-1.05		0.78	0.59-1.03	
BMI (kg/m^2^)						
< 25 (n = 1645)	0.76	0.54-1.06	0.63	0.75	0.54-1.06	0.55
≥ 25 (n = 581)	0.85	0.53-1.36		0.89	0.55-1.44	
Cumulative smoking exposure						
Pack-years < 20 (n = 1679)	0.83	0.60-1.16	0.72	0.81	0.58-1.14	0.64
Pack-years ≥ 20 (n = 547)	0.73	0.45-1.18		0.69	0.41-1.13	
Leisure-time physical activity^c^						
Sedentary (n = 1349)	1.01	0.72-1.42	0.03	1.03	0.72-1.46	0.01
Active (n = 877)	0.55	0.35-0.88		0.49	0.31-0.80	
Fasting (n = 1417)	0.83	0.58-1.18		0.80	0.58-1.16	
BMI (kg/m^2^)						
< 25 (n = 1067)	0.76	0.48-1.18	0.68	0.73	0.46-1.14	0.58
≥ 25 (n = 350)	0.90	0.49-1.64		0.92	0.50-1.70	
Cumulative smoking exposure						
Pack-years < 20 (n = 1059)	0.86	0.54-1.35	0.68	0.81	0.51-1.29	0.74
Pack-years ≥ 20 (n = 358)	0.74	0.42-1.32		0.75	0.41-1.36	
Leisure-time physical activity^c^						
Sedentary (n = 872)	1.17	0.76-1.80	0.008	0.18	0.75-1.86	0.007
Active (n = 545)	0.43	0.22-0.83		0.43	0.22-0.84	

CI, confidence interval; OR, odds ratio.

^a^ Adjusted for age (continuous), sex (male and female), and enrollment area (Shizuoka, Aichi, Shiga, Kyoto, Tokushima, Fukuoka, Saga and Kagoshima.

^b^ Adjusted for age, sex, enrollment area, BMI (continuous), fat energy ratio (continuous), smoking habits (never, light, moderate, and heavy), leisure-time physical activity (sedentary, moderate active, and very active), and blood sampling time from last meal (≥ 12 h, 8 h to < 12 h, 4 h to < 8 h, and 2 h to < 4 h).

^c^ Sedentary, metabolic equivalent (MET)-minutes/week < 600; Active, MET-minutes/week ≥ 600.

All subjects were stratified into two subgroups by BMI, pack-years of smoking, or leisure-time physical activity. There was no significant association between the polymorphism and hypertriglyceridemia in the subgroups stratified by BMI or pack-years of smoking. However, the likelihood of hypertriglyceridemia among subjects with the C allele was significantly lower than those without it in the active group. In addition, gene-environment interactions between the polymorphism (TC + CC vs. TT) and leisure-time physical activity (active vs. sedentary) for hypertriglyceridemia were also significant in both the total and fasting group. When stratified by sex, the interaction was significant only for females in the fasting group (*P* = 0.04), and not significant for both males and females in the total group.

### Serum triglyceride levels according to leisure-time physical activity

**Figure 1:**
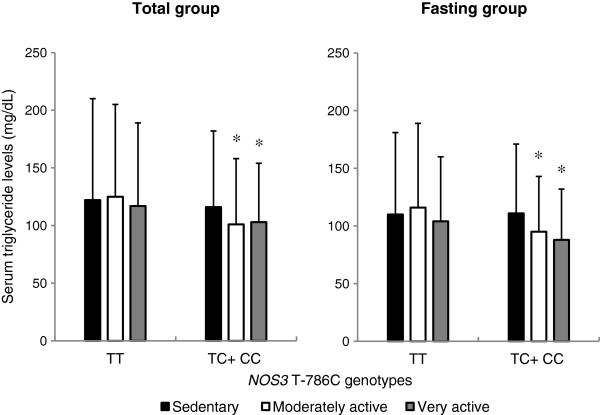
**Serum triglyceride levels according to leisure-time physical activity by the *NOS3 *T-786C genotype.** Serum triglyceride levels are presented as mean ± SD. Trends of serum triglyceride levels across leisure-time physical activity classes, namely, sedentary, moderately active, and very active, and differences in serum triglyceride levels between the classes were tested after adjustment for age (continuous), sex (male and female), enrollment area (Shizuoka, Aichi, Shiga, Kyoto, Tokushima, Fukuoka, Saga, and Kagoshima), BMI (continuous), fat energy ratio (continuous), smoking habits (never, light, moderate, and heavy), and blood sampling time from last meal (≥ 12 h, 8 h to < 12 h, 4 h to < 8 h, and 2 h to < 4 h) by multiple linear regression analysis. Values of serum triglyceride levels were log-transformed before analysis was carried out. * *P* < 0.05 compared to the sedentary group in each genotype group.

Figure 1 shows serum triglyceride levels according to leisure-time physical activity in the TT and TC + CC genotype groups. Serum triglyceride levels across leisure-time physical activity classes decreased significantly only in the TC + CC genotype group (111 ± 60 mg/dL for sedentary, 95 ± 48 mg/dL for moderately active, 88 ± 44 mg/dL for very active, *P* for trend = 0.008 in the fasting group), but not in the TT genotype group. Moreover, in the TC + CC genotype group, both the moderately and very active groups had significantly lower serum triglyceride levels than the sedentary group in the total group (*P* = 0.005 for moderately active and *P* = 0.02 for very active) and the fasting group (*P* = 0.04 for moderately active and *P* = 0.03 for very active). No significant difference was seen in the TT genotype group.

## Discussion

The present study found that gene-environment interactions between the *NOS3* T-786C polymorphism and leisure-time physical activity on serum triglyceride levels were significant, and that both the moderately and very active groups had significantly lower serum triglyceride levels than the sedentary group in the TC + CC genotype group, but not in the TT genotype group. This suggests that the presence of the C allele enhances the beneficial effect of leisure-time physical activity on serum triglyceride levels. An intervention study showed that the presence of the C allele did not affect the beneficial effect of exercise in reducing serum triglyceride levels among postmenopausal women (n = 49) [19], but the sex difference of this effect was unclear in the current study.

The genotype distribution of the current study subjects was very similar to that described previously in 233 Japanese (83.3%, 15.0%, and 1.7% for the TT, TC, and CC genotypes, respectively) [10]. Compared with Caucasians, C allele carriers were much less common in Japanese (32.0%, 52.0%, and 16.0% for the TT, TC, and CC genotypes in Caucasians, respectively) [20].

Recently, the role of triglycerides in atherosclerotic diseases was controversial because of the large variation in triglyceride measurements, and the strong inverse relationship between HDL cholesterol and triglycerides [21]. However, large meta-analyses of prospective studies have reported that triglycerides are an independent risk factor for coronary heart disease in the general Western populations, and Asian and Pacific populations [22,23]. Triglycerides are not directly atherogenic, but represent an important biomarker of cardiovascular disease risk because of their association with atherogenic remnant particles and apolipoprotein C-III, a lipoprotein lipase (LPL) inhibitor [24]. Therapy with fibrates, most selective triglyceride-reducing drugs, was reported to produce a 10% risk reduction for major cardiovascular events and a 13% relative risk reduction for coronary heart disease [25].

Triglycerides are frequently associated with lifestyle factors, one of which is exercise. Aerobic exercise was reported to cause a 15% to 20% reduction in serum triglycerides when triglyceride levels were over 150 mg/dL [24]. In rats, exercise induces an increase in LPL mass and total and heparin-releasable LPL activity in white skeletal muscle [26]. Interestingly, these beneficial effects of exercise may involve NO [27]. *In vitro*, shear stress of bovine aortic endothelial cells increased the eNOS transcriptional rate and upregulated eNOS mRNA levels [28]. An experiment using Goto-Kakizaki rats (a model of type 2 diabetes) showed that exercise increased both the total eNOS expression and the dimer:monomer ratio in the left ventricle and induced significant increases in NO production and concomitant decreases in eNOS-dependent superoxide production [29].

NO can lower serum triglyceride levels by three mechanisms. Firstly, NO can promote triglyceride metabolism through an increase in blood flow and insulin to tissues [30]. Insulin and its counterregulation have been reported to play a central role in triglyceride metabolism by suppression of hepatic production and secretion of triglyceride-rich lipoproteins [31] and stimulation of LPL activity [32]. Secondly, NO can decrease free fatty acid concentration for the synthesis of triglycerides by promoting the formation of new metabolically active mitochondria [33] and activating carnitine palmitoyltransferase 1, an enzyme which transports activated fatty acids into mitochondria for oxidation [34]. Thirdly, NO can suppress the synthesis of triglycerides in liver by inhibiting the de novo fatty acid synthesis through an adenosine monophosphate-activated protein kinase-dependent inhibition of the activity of acetyl-CoA carboxylase, which catalyzes the carboxylation of acetyl-CoA to malonyl-CoA, a key substrate in chain elongation during fatty acid biosynthesis [34].

To date, there has been no experimental study that has shown the difference in responsiveness to exercise in NO production among the T-786C genotypes. It has been reported that a shear stress responsive element is located near the promoter region of *NOS3* between nucleotides −1600 and −779 [35], but the question of whether the polymorphism modifies the effect of physical activity on NO production is controversial. An intervention study showed that six months of aerobic exercise increased the bioavailability of NO only in subjects with the C allele [36]. In contrast, another study showed that the increase in NO production in response to six months exercise was greater in women without the C allele as compared with those with it [37].

In addition to exercise, the polymorphism has been reported to modulate the effect of statins, which are 3-hydroxy-3-methyl-glutaryl-CoA reductase inhibitors [38]. An *in vitro* experiment demonstrated that fluvastatin augmented eNOS transcriptional activity in human umbilical vein endothelial cells (HUVECs) containing the CC genotype more powerfully than those with the TT genotype. This effect was due to a decrease in gene expression of replication protein A1 (RPA1). RPA1 specifically binds to the C allele and significantly suppresses the transcriptional activity of *NOS3*[39]. This finding was supported by an epidemiological study that showed atorvastatin treatment increased whole blood nitrite concentrations and attenuated oxidative stress in the CC genotype group, but not in the TT genotype group [40]. Future studies investigating the effect of exercise on RPA1 expression by the T-786C genotype is eagerly anticipated.

The current study had several limitations. To begin with, the examination was limited to only one *NOS3* polymorphism and did not consider combinations with other *NOS3* polymorphisms, as well as polymorphisms of other genes that are associated with serum triglyceride levels. Next, there may have been differences among the institutions in measurement methods concerning clinical data. However, such differences may have had a limited impact on data analysis as enrollment area was added to the adjustment factors. Finally, physical activity of subjects was taken into account only during their leisure time, not all the time, and the effect of physical activity outside of leisure time may have been confounding.

In conclusion, the present study suggests that the *NOS3* T-786C polymorphism modifies the effect of leisure-time physical activity on serum triglyceride levels. Tailoring prevention of hypertriglyceridemia on the basis of the T-786C genotypes could be achieved, provided that further studies, including cohort and intervention studies, validate the results of this cross-sectional study.

## Competing interests

The authors declare that they have no competing interests.

## Authors’ contributions

TH was responsible for the complete analysis of the data and for preparing the tables and the manuscript. NH, HT, and KW participated in all aspects of the project, including study design, molecular genetic studies, statistical analysis, and manuscript preparation. MN, SK, and GY were responsible for data management, and carried out the molecular genetic studies. SS, YK, HN, TI, KO, KA, MS, HI, and HM acted as principal investigators for the J-MICC Study, and contributed to data acquisition. MK carried out the genotyping. All authors have read and approved the manuscript.
